# Enhanced neuroinflammation mediated by DNA methylation of the glucocorticoid receptor triggers cognitive dysfunction after sevoflurane anesthesia in adult rats subjected to maternal separation during the neonatal period

**DOI:** 10.1186/s12974-016-0782-5

**Published:** 2017-01-07

**Authors:** Yangzi Zhu, Yu Wang, Rui Yao, Ting Hao, Junli Cao, He Huang, Liwei Wang, Yuqing Wu

**Affiliations:** 1 Jiangsu Province Key Laboratory of Anesthesiology, Xuzhou Medical University, 209 Tongshan, Xuzhou, 221004 People’s Republic of China; 2 Department of Anesthesiology, Xuzhou Central Hospital, 199 Jiefang South Road, Xuzhou, People’s Republic of China; 3 Department of Anesthetic Pharmacology, Xuzhou Medical University, Xuzhou, People’s Republic of China

**Keywords:** Sevoflurane, Neuroinflammation, Pro-inflammatory factors, Cognitive dysfunction, Glucocorticoid receptor, DNA methylation

## Abstract

**Background:**

Mounting evidence indicates that children who experience abuse and neglect are prone to chronic diseases and premature mortality later in life. One mechanistic hypothesis for this phenomenon is that early life adversity alters the expression or functioning of the glucocorticoid receptor (GR) throughout the course of life and thereby increases sensitivity to inflammatory stimulation. An exaggerated pro-inflammatory response is generally considered to be a key cause of postoperative cognitive dysfunction (POCD). The aim of this study was to examine the effects of early life adversity on cognitive function and neuroinflammation after sevoflurane anesthesia in adult rats and to determine whether such effects are associated with the epigenetic regulation of GR.

**Methods:**

Wistar rat pups were repeatedly subjected to infant maternal separation (early life stress) from postnatal days 2–21. In adulthood, their behavior and the signaling of hippocampal pro-inflammatory factors and nuclear factor-kappa B (NF-κB) after sevoflurane anesthesia were evaluated. We also examined the effects of maternal separation (MS) on the expression of GR and the DNA methylation status of the promoter region of exon 1_7_ of GR and whether behavioral changes and neuroinflammation after anesthesia were reversible when the expression of GR was increased by altering DNA methylation.

**Results:**

MS induced cognitive decline after sevoflurane inhalation in the Morris water maze and context fear conditioning tests and enhanced the release of cytokines and the activation of astrocyte intracellular NF-κB signaling induced by sevoflurane in the hippocampus of adult rats. Blocking NF-κB signaling by pyrrolidine dithiocarbamate (PDTC) inhibited the release of cytokines. MS also reduced the expression of GR and upregulated the methylation levels of the promoter region of GR exon 1_7_, and such effects were reversed by treatment with the histone deacetylase inhibitor trichostatin A (TSA) in adult rats. Moreover, TSA treatment in adult MS rats inhibited the overactivation of astrocyte intracellular NF-κB signaling and the release of cytokines and alleviated cognitive dysfunction after sevoflurane anesthesia.

**Conclusions:**

Early life stress induces cognitive dysfunction after sevoflurane anesthesia, perhaps due to the aberrant methylation of the GR gene promoter, which reduces the expression of the GR gene and facilitates exaggerated inflammatory responses.

## Background

Postoperative cognitive dysfunction (POCD) is a relatively common and well-known complication in surgical patients. The occurrence of POCD is associated with increased mortality, risk of withdrawal from the labor market, and dependency on social transfer payments [1, 2]. However, despite enormous research efforts in recent decades, the pathogenesis of POCD remains obscure. Mounting evidence has revealed that inflammation plays a key role in the disease process [3–5]. Peripheral inflammation due to surgical trauma and the release of accompanying systemic inflammatory mediators have been shown to influence inflammatory processes of the central nervous system, triggering the activation of neurogliocytes and the concurrent endogenous production of pro-inflammatory cytokines [6–9]. Volatile anesthetics, particularly isoflurane and sevoflurane, directly increased the production of pro-inflammatory cytokines in the brains of mice and impaired the acquisition of spatial memory in aged rats [10–12]. In addition to surgical trauma and anesthetics, advanced age is one of the main risk factors for the development of POCD [13]. Aging has been associated with an exacerbated inflammatory response in the normal aged brain when the immune system is irritated [14, 15], which may increase susceptibility to POCD in the aged [16–18]. However, the occurrence of POCD exhibits pronounced individual differences in the elderly, and POCD is also observed in younger and adult patients [2].

Accumulating evidence indicates that early life adversity is associated with an increased risk for various long-term mental and physical diseases, including depression, anxiety, autoimmune disorders, diabetes, hypertension, cardiovascular diseases, cancers, and premature mortality [19–25]. The consequences of early life adversity can last a lifetime, even when individuals’ life situations improve after experiencing early negative life events [26, 27]. A hypothesis concerning the mechanism of susceptibility to chronic diseases is that early life adversity induces glucocorticoid insensitivity and exaggerates inflammatory responses to injury or infection [28]. Several studies have shown that early life adversity results in an increased methylation of the glucocorticoid receptor (GR) gene promoter and reduced GR expression in rodents and humans [29–31], which may lead to glucocorticoid feedback resistance and GR desensitization [28, 32]. It is generally accepted that GR signaling regulates the inflammatory response to noxious stimuli, and evidence has supported this hypothesis. Adult individuals who experienced early life adversity were shown to have elevated levels of C-reactive protein (CRP) and pro-inflammatory cytokines (interleukin (IL)-1, IL-6, and tumor necrosis factor (TNF)-α) compared with participants without early life stress [33–36]. Furthermore, an animal study showed that early life stress enhanced the production of pro-inflammatory cytokines in response to viral infection [37]. Thus, will early life adversity amplify the neuroinflammation induced by volatile anesthetics or surgeries and increase the risk of POCD?

The aim of this study was to examine whether early life adversity induces cognitive decline and exaggerates neuroinflammation after sevoflurane anesthesia in adult rats. Moreover, we examined the effects of early life adversity on the expression of GR and the methylation of GR gene promoters and whether behavioral changes and neuroinflammation after anesthesia were reversible if GR expression was increased by altering DNA methylation.

## Methods

### Maternal separation model

All experimental procedures involving animals were approved by the Animal Care and Use Committee of Xuzhou Medical University (Xuzhou, Jiangsu Province, China). Wistar rats were obtained from the Experimental Animal Center of the Xuzhou Medical University. Male and female rats were housed together in standard rat cages, each containing one male and two females. Each female rat was housed separately after pregnancy. Animals were maintained under standard laboratory conditions, with a 12-h light/dark cycle (lights on at 6:00 a.m.), a room temperature of 23 ± 1 **°**C, and food and water provided ad libitum. The pups remained undisturbed at birth (postnatal day 0, P0). Half of the male pups from each litter were randomly assigned to undergo maternal separation (MS). The other half of the male pups and all female pups were kept with their dam the entire time. The separated pups were removed from the home cage and placed in a warm standard laboratory cage for 6 h per day (7:00–10:00 and 13:00–16:00) from P1 to P21 [38]. The pups were returned to the home cage and remained with their dam the rest of the time. Following weaning, all pups were group-housed at P22 (6–7 per cage) according to gender and whether they had been subjected to MS or not. We did not observe any differences in body weight between MS rats and normal rats (data not shown). Subsequent experiments were performed when the animals reached adult age (85–90 days old).

### Rats anesthesia and treatment

Rats received 3% sevoflurane mixed with pure oxygen for 2 h in anesthetizing chambers. Control groups received pure oxygen for 2 h in identical chambers. The anesthesia with 3% sevoflurane mixed with pure oxygen for 2 h was clinically relevant and did not cause significant changes in blood pressure and blood gas compared with the control group (data not shown). Anesthetizing chambers were placed on a heating pad, and the rat’s body temperature was maintained at 37 ± 0.5 °C. The concentrations of sevoflurane and oxygen were measured continuously (Drager, Lubeck, Schleswig-Holstein, Germany). Anesthesia was terminated by discontinuing sevoflurane and by administering pure oxygen until the animal regained its righting reflex. To evaluate the role of the nuclear factor-kappa B (NF-κB) signaling pathway, pyrrolidine dithiocarbamate (PDTC; 100 mg/kg) [39], an NF-κB inhibitor, was given to rats intraperitoneally 30 min before exposure to sevoflurane. For intervention studies, the histone deacetylase inhibitor trichostatin A (TSA) was used for the epigenetic regulation of the glucocorticoid receptor. Rats were implanted with a stainless steel guide cannula (22 gauge, RWD, China) directed toward the left lateral ventricle (1.6 mm lateral to the midline, 1.0 mm posterior to the bregma, and 4.0 mm below the surface of the dura). Other procedures were performed as described in previous studies [29]. A volume of 2 μl of TSA (100 ng/ml in DMSO) was infused using a micro-syringe through the infusion cannula for at least 1 min. Rats received a single infusion daily for seven consecutive days before exposure to sevoflurane.

### Morris water maze tests

The Morris water maze (MWM) was a cylindrical, black-painted pool (1.5 m in diameter, 0.6 m in height) filled with water (0.3 m deep, 24 ± 1 °C) and divided into four virtual quadrants, with one starting point in each quadrant. A black-painted platform (10 cm diameter, 1 cm below water surface) was placed in the determinate quadrant. The experimental room contained cues that remained unchanged throughout the study. The movements of the rats were recorded by a video-tracking/computer-digitizing system (Shanghai Jiliang Software Technology Co., Ltd., Shanghai, China). The P86 rats were tested in the MWM four times per day for 4 days (acquisition tests). Each rat was gently placed in the water at one of the four starting points (in a random order) along the water maze perimeter with its face toward the wall of the pool. Rats were given 120 s to find the platform and were then left on the platform for 30 s. If the rat did not find the hidden platform within 120 s, the researcher would gently guide it to the platform, and its escape latency to find the platform was then marked as 120 s. At the end of the MWM training (P90), the platform was removed and the animals were allowed to search in the pool for 120 s (probe test); the number of crossings over the target area and the search time in the target quadrant were recorded [40]. Animals that performed MWM tests were not used in other tests.

### Context fear conditioning tests

Fear conditioning experiments were performed in an acrylic conditioning chamber with a grid floor composed of 19 stainless steel bars. A ventilation fan supplied background noise (65 db), and overhead infrared lights were left on. An infrared camera was located on the walls to monitor the rats’ freezing behavior. P86 rats were given 5 min to acclimate to the chamber and were then presented with one tone (2.2 kHz and 96 dB for 30 s). During the last 2 s of the tone, the rats were given a single shock (2.0 mA, 2 s). After the shock, the rats stayed in the chamber for 30 s and were then placed back into their housing area. Memory retention was assessed at 48 h post-conditioning. P88 rats were returned to the original chamber and allowed to stay there for 3 min. The chamber was maintained in the same context as when the rats were conditioned but without a footshock or tone. Freezing time during this period was recorded as a measure of contextual fear memory [41]. Animals that underwent context fear conditioning tests were not used in other tests.

### Hippocampus tissue preparation

Immediately after undergoing anesthesia with 10% chloral hydrate (0.3 ml/100 g, i.p.), all animals were sacrificed by decapitation. The hippocampus was rapidly removed, frozen in liquid nitrogen, and then stored at −80 °C until further processing. For enzyme-linked immunosorbent assay and Western blot analysis, the harvested hippocampus tissues were homogenized on ice using radioimmunoprecipitation assay (RIPA) lysis buffer supplemented with protease inhibitors (Beyotime Institute of Biotechnology, Haimen, Jiangsu, China). The lysates were collected and centrifuged at 12,000 rpm for 15 min to extract total proteins. The EpiQuik™ Nuclear Extraction Kit (Epigentek, Brooklyn, NY, USA) was used for the preparation of nucleoproteins from the hippocampal tissues according to the manufacturer’s instructions. The supernatant was quantified for total protein using the bicinchoninic acid (BCA) protein assay kit (Beyotime Institute of Biotechnology, Haimen, Jiangsu, China).

### Western blot analysis

Western blotting was used to determine the expression of GR in the total protein extract and NF-κB p65 in the nuclear extract from the hippocampal tissues. Equal amounts of protein were loaded and separated by sodium dodecyl sulfate-polyacrylamide gel electrophoresis (SDS-PAGE) and transferred to nitrocellulose membranes (Millipore Corporation, Billerica, MA). The membranes were blocked in 5% nonfat milk for 2 h at room temperature and then incubated overnight at 4 °C with rabbit anti-GR (1:200, Santa Cruz Biotechnology, CA, USA), rabbit anti-NF-κB p65 (1:500, Abcam, Cambridge, UK), and mouse anti-β-actin (1:1000, Sigma-Aldrich). After the incubation, the membranes were washed three times with phosphate-buffered saline (PBS, pH 7.4) containing 0.3% Triton X-100 (PBS-T) and incubated with corresponding secondary antibodies conjugated with horseradish peroxidase (1:500, ZSGB-BIO, Beijing, China) for 2 h at room temperature. The protein signals were finally visualized using an enhanced BCIP/NBT Alkaline Phosphatase Color Development Kit (Beyotime Biotechnology, Inc., Haimen, Jiangsu, China).

### Enzyme-linked immunosorbent assay

TNF-α, IL-1β, and IL-6 levels were quantified using rat-specific enzyme-linked immunosorbent assay (ELISA) kits (Xitang Bio-tech. Co., Ltd, Shanghai, China) according to the manufacturer’s instructions. The optical density of each well was determined using a microplate reader at 450 nm. Cytokine concentrations were calculated using standard curves generated using recombinant TNF-α, IL-1β, and IL-6.

### Immunofluorescent staining

Rats were anesthetized with 10% chloral hydrate (0.3 ml/100 g, i.p.) and perfused transcardially with 200 ml of 0.9% saline followed by 300 ml of 4% paraformaldehyde in 0.1 M phosphate buffer (pH 7.4). The brain tissues were removed and postfixed in 4% paraformaldehyde overnight and cryoprotected in 30% sucrose. Thirty-micron-thick frozen sections from the rat brains were cut using a freezing microtome and serially collected throughout the hippocampus. Free-floating tissue sections were rinsed three times with PBS-T. The tissue sections were incubated with 10% normal donkey serum in PBS-T for 2 h, followed by incubation with rabbit anti-p-NF-κB p65 (1:500, Cell Signaling Technology, Inc., Beverly, MA) and mouse anti-glial fibrillary acidic protein (GFAP) monoclonal antibody (1:600, Abcam, Cambridge, UK) at 4 °C for 24 h. After three 5-min rinses in PBS, the sections were incubated with donkey anti-rabbit IgG conjugated to Alexa Fluor® 488 and donkey anti-mouse IgG conjugated to Alexa Fluor® 594 (1:500, Life Technologies, Carlsbad, CA, USA) in the dark for 2 h at 37 °C. Fluorescence intensity was visualized under a confocal microscope (FV1000, Olympus Corp., Tokyo, Japan). The intensity on four slides (three to four sections per slide) was averaged for each animal and then normalized by that of the control group.

### Quantitative reverse transcriptase polymerase chain reaction

Quantitative reverse transcriptase polymerase chain reaction (qRT-PCR) was used to assess the mRNA levels of GR in rat hippocampal tissue. Total RNA was extracted using a TRIzol reagent kit (Invitrogen, USA) and transcribed into cDNA using a high-capacity cDNA reverse transcription kit (Applied Biosystems, Foster City, USA). The PCR primers for GR were 5′-TAGGTGGGCGTCAAGTGATT-3′ (forward) and 5′-GATCAGGAGCAAAGCAGAGC-3′ (reverse). The primers for GAPDH were 5′-CAAGGTCATCCATGACAACTTTG-3′ (forward) and 5′-GTCCACCACCCTGTTGCTGTAG-3′ (reverse). Real-time PCR analysis was performed using the Roche LightCycler® 480 detection system using a SYBR® Select Master Mix Kit (Life Technologies, Carlsbad, CA, USA). The relative expression levels of GR were normalized to GAPDH.

### DNA methylation assay

DNA was extracted from the hippocampal tissue using the genome extraction kit (Generay Biotechnology, Shanghai, China). Sodium bisulfite modification of DNA was performed using the EpiTect Bisulfite kit (Qiagen, Venlo, Netherlands) according to the manufacturer’s protocol. PCR amplification was carried out in a 50-μl reaction volume containing 25 μl of PCR master mix (Qiagen, Germany), 2 μl of each primer, 2.5 μl of bisulfite-modified DNA, and H_2_O. The thermocycler protocol was as follows: a 4-min denaturation at 95 °C; 40 cycles of 30-s denaturation at 95 °C, a 30-s annealing period at 55 °C, and a 40-s extension at 72 °C; and a final 5-min extension at 72 °C. The PCR product (285 bp) was used as a template for subsequent nested PCR reactions. The PCR product (177 bp) was purified and separated on a 2% agarose gel. The targeted DNA fragment was extracted from the gel. Following elution in Tris buffer, the PCR product was then subcloned into the pTG19-T vector (Generay Biotechnology) according to the manufacturer’s protocol. Ten plasmids containing the exon 1_7_ GR promoter DNA fragment were screened and sequenced per animal.

### Statistical analysis

All of the data are presented as the mean ± SD of independent experiments. For the escape latency data, repeated-measures two-way ANOVAs between groups were conducted. The post hoc Bonferroni test was used to compare the differences in escape latency between groups on each day of the MWM. One-way ANOVA followed by the Student–Newman–Keuls post hoc test or Student’s two-sample *t* test was performed for the other behavioral tests, Western blotting, ELISA, and methylation of all CpG sites within exon 1_7_ of GR. Statistical analysis was performed using SPSS 16.0 (SPSS, Chicago IL) or GraphPad Prism 5.0 for Windows (GraphPad Software, Inc., San Diego, CA). *P* values <0.05 were considered to be statistically significant.

## Results

### Sevoflurane anesthesia induced cognitive impairment in adult maternal separation rats

**Fig. 1:**
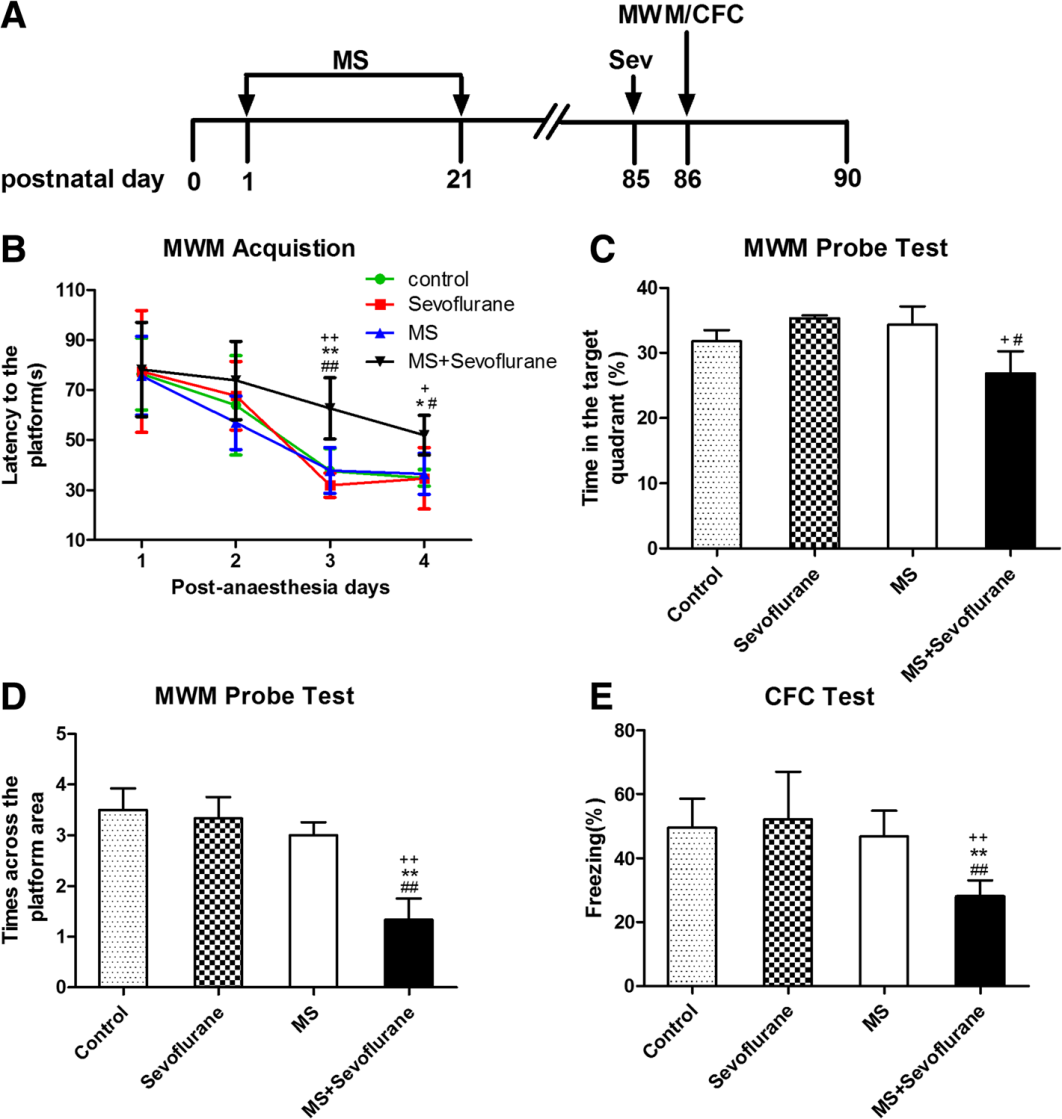
The effect of neonatal maternal separation on cognitive function after sevoflurane anesthesia in adult rats. **a** Schematic illustration of the experimental timeline. *MS* maternal separation, *Sev* anesthesia with 3% sevoflurane for 2 h, *MWM* Morris water maze, *CFC* context fear conditioning. **b** Anesthesia with 3% sevoflurane for 2 h in MS rats increased the escape latency in the acquisition phase of the MWM test on the third and fourth days after sevoflurane anesthesia. There was no statistically significant interaction of time and group between groups. Analysis was performed with repeated-measures two-way ANOVA. **c** The time in the target quadrant in the MS + sevoflurane group was less than those in the sevoflurane group and MS group by the MWM probe test. **d** The number of crossings of the platform area was lower in the MS + sevoflurane group than in the other three groups in the MWM probe test. **e** MS rats subjected to sevoflurane anesthesia exhibited reduced freezing to context when compared with normal rats that received sevoflurane anesthesia and MS rats without anesthesia. ^*^ *P* < 0.05, ^**^ *P* < 0.01 versus control; ^+^ *P* < 0.05, ^++^ *P* < 0.01 versus sevoflurane; ^#^ *P* < 0.05, ^##^ *P* < 0.01 versus MS. *Error bars* represent the means ± SD (*n* = 10). Statistical analyses were performed using a one-way ANOVA followed by Student–Newman–Keuls post hoc test

To elucidate the effect of MS on cognitive function after sevoflurane inhalation in adult rats, we conducted MWM tests and context fear conditioning (CFC) tests, which are widely used to evaluate hippocampus-dependent spatial reference learning and memory in rodents [42, 43]. A schematic illustration of the experimental timeline is shown in Fig. 1a. The comparison of the time that each rat took to reach the platform during reference training (escape latency) showed that there was no statistically significant interaction of time and group between groups in the acquisition phase of the MWM tests (*F* = 1.567, *P* = 0.145). The factors of days (*F* = 29.488, *P* < 0.001) and groups (*F* = 4.714, *P* = 0.011) were statistically significant. The post hoc Bonferroni test showed that the MS rats that received sevoflurane anesthesia had a longer escape latency than the normal rats; the normal rats that received sevoflurane anesthesia and the MS rats without anesthesia on the third or fourth days after sevoflurane anesthesia (Fig. 1b; post-anesthesia day 3, *P* = 0.001 MS + sevoflurane versus control, *P* < 0.001 MS + sevoflurane versus sevoflurane, and *P* = 0.001 MS + sevoflurane versus MS; post-anesthesia day 4, *P* = 0.014 MS + sevoflurane versus control, *P* = 0.013 MS + sevoflurane versus sevoflurane, and *P* = 0.030 MS + sevoflurane versus MS). Reference memory was assessed using the MWM probe test on the fifth day. The time spent in the target quadrant in the MS + sevoflurane group was less than that in MS group and sevoflurane group (Fig. 1c; *P* = 0.020 MS + sevoflurane versus sevoflurane and *P* = 0.037 MS + sevoflurane versus MS). In addition, the number of crossings of the platform area was lower in the MS + sevoflurane group than in the other groups (Fig. 1d; *P* = 0.001 MS + sevoflurane versus control, *P* = 0.002 MS + sevoflurane versus sevoflurane, and *P* = 0.007 MS + sevoflurane versus MS). MS rats showed a significant cognitive decline after sevoflurane anesthesia in MWM tests. The results of the CFC tests were similar to those of the MWM tests; MS rats that received sevoflurane anesthesia exhibited reduced immobility (freezing) in the CFC test (Fig. 1e; *P* = 0.001 MS + sevoflurane versus control, *P* < 0.001 MS + sevoflurane versus sevoflurane, and *P* = 0.004 MS + sevoflurane versus MS). No statistically significant differences were observed among the control group, sevoflurane group, and MS group in the MWM and CFC tests.

### Maternal separation enhanced the release of cytokines and activation of astrocytes and NF-κB signaling induced by sevoflurane in the hippocampus

**Fig. 2:**
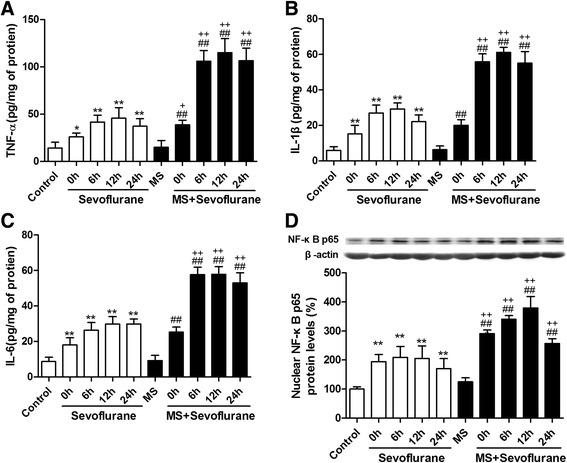
MS enhanced the release of cytokines and the expression of nuclear NF-κB p65 induced by sevoflurane in the hippocampus. **a** Sevoflurane anesthesia for 2 h increased the levels of TNF-α at 0, 6, 12, and 24 h after anesthesia in both control and MS rats. The levels of TNF-α in MS rats were higher than that in control rats at each time point after anesthesia. The changes in the levels of IL-1β (**b**), IL-6 (**c**), and nuclear NF-κB p65 (**d**) in the hippocampus were similar to those of TNF-α. ^*^ *P* < 0.05, ^**^ *P* < 0.01 versus control; ^##^ *P* < 0.01 versus MS; ^+^ *P* < 0.05, ^++^ *P* < 0.01 MS + sevoflurane versus sevoflurane (at the same time point). *Error bars* represent the means ± SD (*n* = 6). Statistical analyses were performed by one-way ANOVA followed by Student–Newman–Keuls post hoc test

The above findings suggested that sevoflurane anesthesia in MS rats might induce cognitive impairment; therefore, we continued to investigate the underlying mechanisms. It has been reported that pro-inflammatory cytokines, such as TNF-α, IL-1β, and IL-6, are associated with cognitive impairment [6–9]. Therefore, we detected the levels of TNF-α, IL-1β, and IL-6 in the hippocampi of rats after sevoflurane anesthesia. An ELISA for TNF-α showed that 3% sevoflurane anesthesia for 2 h increases the levels of TNF-α at 0, 6, 12, and 24 h after anesthesia both in control and MS rats, but the levels of TNF-α in MS rats were markedly higher than in control rats at each time point after anesthesia (Fig. 2a). The changes in the levels of IL-1β and IL-6 in the hippocampus were similar to those of TNF-α (Fig. 2b, c).

Previous studies have shown that inhaled anesthetic can activate the NF-κB signaling pathway [44], which is a core signaling pathway associated with the expression of pro-inflammatory cytokines. To examine whether the observed changes in inflammatory mediators are related to the NF-κB signaling pathway, we determined the expression of NF-κB p65 in the nuclear extracts from the hippocampal tissues. Western blot analysis showed that 3% sevoflurane anesthesia for 2 h increases the levels of nuclear NF-κB p65 protein at 0, 6, 12, and 24 h after anesthesia both in control and MS rats, but the levels of nuclear NF-κB p65 in MS rats were markedly higher than that in control rats at each time point after anesthesia (Fig. 2d).

**Fig. 3:**
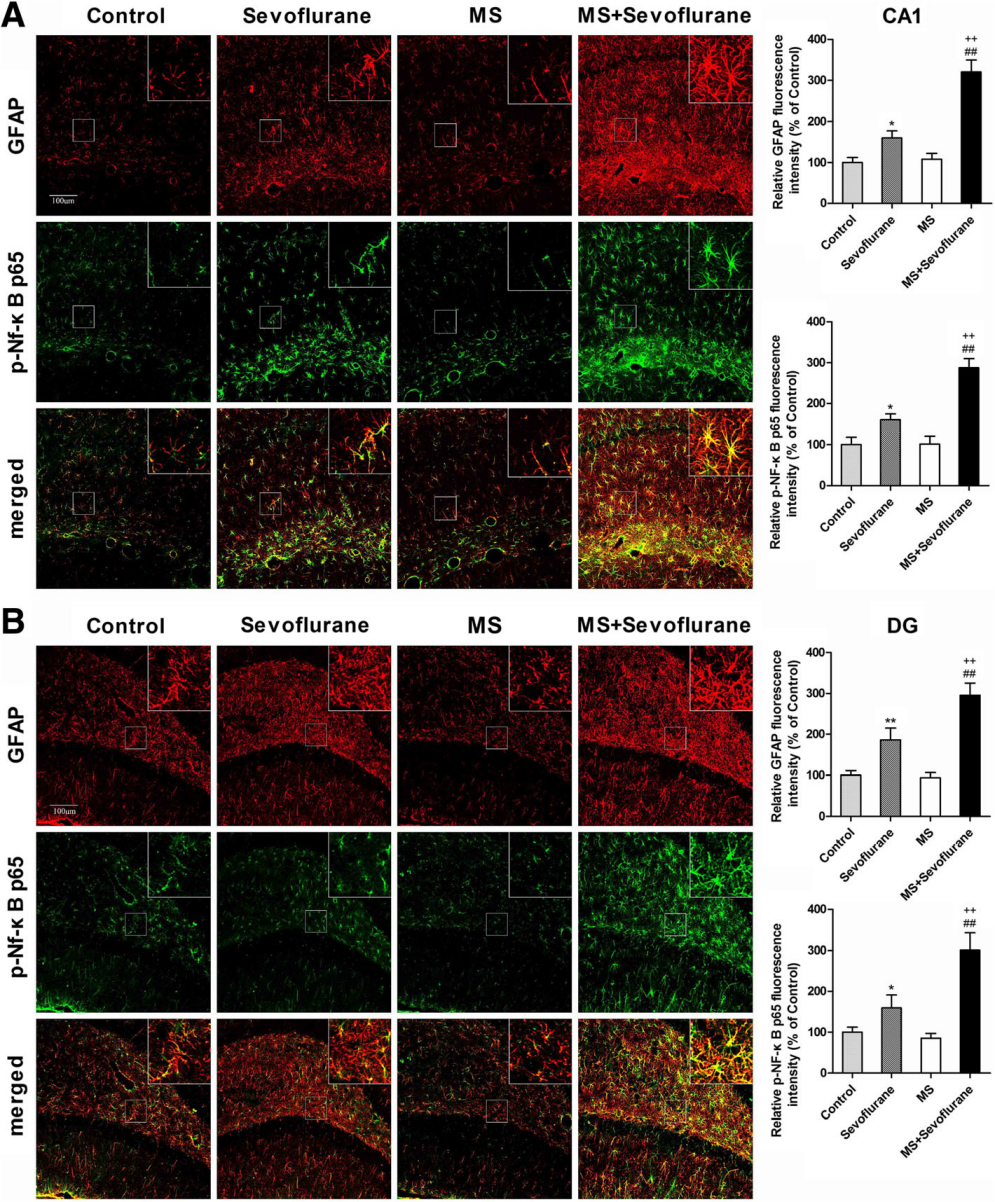
MS enhanced the activation of astrocytes and the astroglial NF-κB signaling pathway induced by 3% sevoflurane anesthesia for 2 h in the hippocampus. **a** Sevoflurane anesthesia increased the fluorescence intensity of glial fibrillary acidic protein (GFAP) and phosphorylated nuclear factor-kappa B (p-NF-κB p65) in the CA1 area of the hippocampus at 12 h after anesthesia in both the sevoflurane group and MS + sevoflurane group, and the fluorescence intensity of GFAP and p-NF-κB p65 in the MS + sevoflurane group was obviously higher than that in the sevoflurane group. Double immunofluorescence staining showed that p-NF-κB p65 (*green*) mainly co-localized (*merged*) with GFAP-positive reactive astrocytes (*red*) in the CA1 area of the hippocampus. **b** The changes in the fluorescence intensity of GFAP and p-NF-κB p65 in the DG area of the hippocampus were similar to those in the CA1 area of the hippocampus. ^*^ *P* < 0.05, ^**^ *P* < 0.01 versus control; ^##^ *P* < 0.01 versus MS; ^++^ *P* < 0.01 MS + sevoflurane versus sevoflurane. *Error bars* represent the means ± SD (*n* = 6). Statistical analyses were performed by one-way ANOVA followed by Student–Newman–Keuls post hoc test. *CA1* cornu ammonis 1, *DG* dentate gyrus; *bar* = 100 μm

The glia is the main source of inflammatory cytokines in the central nervous system. It has been reported that rats with POCD had increased levels of pro-inflammatory cytokines and astrocyte activation [9]. Activation of the astroglial NF-κB signaling pathway can regulate neuroinflammation because it drives the transcription of several pro-inflammatory molecules [45, 46]. We therefore assessed the effects of sevoflurane anesthesia on astrocytes and the astroglial NF-κB signaling pathway in the hippocampus between control and MS rats. GFAP immunofluorescence was increased 12 h after anesthesia in both the sevoflurane group and the MS + sevoflurane groups (CA1, *P* = 0.040 sevoflurane versus control, *P* < 0.001 MS + sevoflurane versus MS; DG, *P* = 0.008 sevoflurane versus control, *P* < 0.001 MS + sevoflurane versus MS). The fluorescence intensity of GFAP in MS + sevoflurane group rats was markedly higher than that in sevoflurane group rats (Fig. 3a, b; CA1, *P* < 0.001 MS + sevoflurane versus sevoflurane; DG, *P* = 0.003 MS + sevoflurane versus sevoflurane). A similar change in the pattern was seen with phosphorylated nuclear factor-kappa B (p-NF-κB p65) (Fig. 3a, b). We also found that p-NF-κB p65 mainly co-localized with GFAP in astrocytes (Fig. 3a, b) but not in microglia in the hippocampus (data not shown), as revealed by double immunofluorescence staining.

### PDTC suppresses the enhancement effect of maternal separation on NF-κB Activation and the release of pro-inflammatory cytokines induced by sevoflurane in the hippocampus

**Fig. 4:**
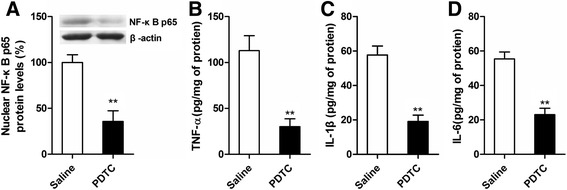
PDTC inhibits the enhancement effect of maternal separation on NF-κB activation and the release of pro-inflammatory cytokines induced by sevoflurane in MS rats. PDTC or vehicle (saline) was given to rats intraperitoneally 30 min before exposure to sevoflurane. **a** PDTC pretreatment decreased the nuclear NF-κB p65 protein levels in the hippocampus 12 h after sevoflurane anesthesia. **b** PDTC pretreatment decreased TNF-α levels in the hippocampus 12 h after sevoflurane anesthesia. **c** PDTC pretreatment decreased IL-1β levels in the hippocampus 12 h after sevoflurane anesthesia. **d** PDTC pretreatment decreased IL-6 levels in the hippocampus 12 h after sevoflurane anesthesia. ^**^ *P* < 0.01 versus saline. *Error bars* represent the means ± SD (*n* = 6). Statistical analyses were performed with Student’s two-sample *t* test

To determine whether the NF-κB signaling pathway was involved in the increase of neuroinflammation after sevoflurane anesthesia in MS rats, we evaluated the effect of the NF-κB inhibitor PDTC on NF-κB activation and the release of pro-inflammatory cytokines 12 h after anesthesia in MS rats. Western blot analysis showed that PDTC pretreatment significantly prevented the enhancement effect of MS on the levels of nuclear NF-κB p65 protein in MS rats (Fig. 4a; *t* = 11.03, *P* < 0.001 PDTC versus saline). Accordingly, an ELISA showed that PDTC also markedly suppressed the increase in hippocampal TNF-α, IL-1β, and IL-6 levels (Fig. 4b–d; TNF-α, *t* = 11.05, *P* < 0.001 PDTC versus saline; IL-1β, *t* = 14.98, *P* < 0.001 PDTC versus saline; IL-6, *t* = 14.57, *P* < 0.001 PDTC versus saline). These data suggest that the abnormal activation of the NF-κB signaling pathway induced by sevoflurane in MS rats may play an important role in exaggerated neuroinflammation.

### Maternal separation alters the DNA methylation status of exon 1_7_ of the GR promoter region and decreases the expression of GR

**Fig. 5:**
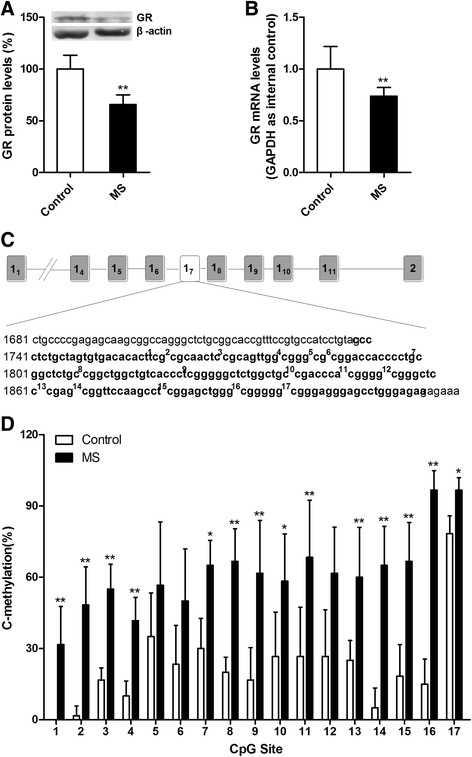
Maternal separation attenuated the expression of GR and increased the percentage of methylated clones within the exon 1_7_ glucocorticoid receptor promoter region from the hippocampal tissues of adult rats. **a** GR protein levels in the hippocampal tissues of MS rats were lower compared with those of control rats. **b** qRT-PCR (GAPDH as an internal control) showed that MS decreased the mRNA levels of GR in the hippocampus compared with the control conditions. **c** The complete sequence map of exon 1_7_ of the GR promoter region (*bold*), including the 17 CpG dinucleotides (*superscript numbers*). **d** The frequency of methylation observed at each CpG site in the exon 1_7_ GR promoter region showed that MS rats have a higher percentage of methylated clones than control rats. ^*^ *P* < 0.05, ^**^ *P* < 0.01 versus control. *Error bars* represent the means ± SD (*n* = 6). Statistical analyses were performed with Student’s two-sample *t* test

Why do MS rats exhibit unusually excessive activation of NF-κB signaling and exaggerated neuroinflammation after sevoflurane anesthesia? The interaction of GR-mediated signaling with NF-κB signaling plays a key role in the regulation of inflammatory responses. It has been reported that early life adversity (such as less maternal care and maltreatment) induces GR desensitization and exaggerates inflammatory responses to injury or infection [28]. We therefore tested the effects of MS on the expression of GR in adult rats. Western blot analysis showed that MS decreased the expression levels of GR proteins in the hippocampus (Fig. 5a; *t* = 4.508, *P* = 0.006 MS versus control). MS-induced decreases of GR levels may be due to decreases in the generation or increases in the degradation of GR. We therefore assessed the effects of MS on the mRNA levels of GR. qRT-PCR analysis showed that MS decreased the mRNA levels of GR in the hippocampi of adult rats (Fig. 5b; *t* = 4.144, *P* = 0.002 MS versus control). To determine whether the DNA methylation of the GR promoter alters the expression levels of GR, we tested the methylation status of 17 CpG sites within the exon 1_7_ GR promoter region from the hippocampal tissue in adult rats. (Fig. 5c). Methylation analysis showed that MS rats have a higher percentage of methylated clones than control rats at most of the 17 CpG dinucleotides in the exon 1_7_ GR promoter region (Fig. 5d).

### Trichostatin A reverses epigenetic alterations and changes in GR expression mediated by MS

**Fig. 6:**
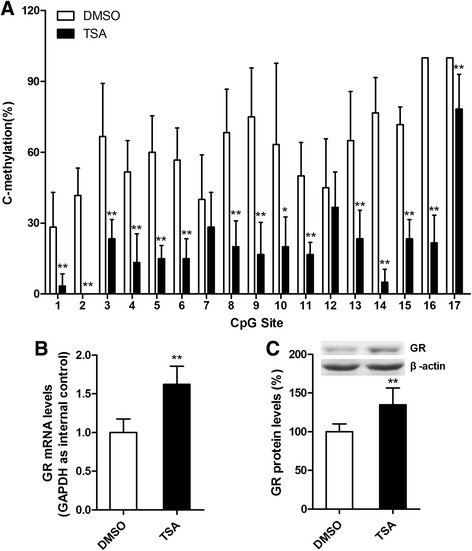
TSA decreased the percentage of methylated clones within the exon 1_7_ GR promoter region and increased the expression of GR in the hippocampal tissues in adult MS rats. A volume of 2 μl of TSA (100 ng/ml in DMSO) was infused into the lateral ventricle of MS rats for seven consecutive days. **a** The frequency of methylation observed at each CpG site in the exon 1_7_ GR promoter region showed that TSA decreased the percentage of methylated clones relative to the DMSO treatment. **b** qRT-PCR (GAPDH as internal control) showed that TSA increased the mRNA levels of GR in MS rats compared with the DMSO treatment. **c** TSA treatment increased the nuclear GR protein levels in the hippocampus of MS rats relative to the DMSO treatment. ^*^ *P* < 0.05, ^**^ *P* < 0.01 versus DMSO. *Error bars* represent the means ± SD (*n* = 6). Statistical analyses were performed with Student’s two-sample *t* test

The above findings suggest that MS influences hippocampal GR expression, possibly through epigenetic alterations. We therefore investigated whether the impact of MS is reversible by altering DNA methylation in adults. Previous research has shown that the histone deacetylase inhibitor TSA may induce the replication-independent demethylation of ectopically methylated genes by increasing histone acetylation. We therefore tested the effects of TSA on the methylation status of the exon 1_7_ GR promoter and the expression of GR in adult MS rats. Methylation analysis of the 17 CpG dinucleotides of the exon 1_7_ GR promoter showed that TSA decreased the percentage of methylated clones relative to the vehicle treatment in adult MS rats (Fig. 6a). qRT-PCR analysis showed that TSA increased the mRNA levels of GR in the hippocampi of MS rats (Fig. 6b; *t* = 5.230, *P* < 0.001 DMSO versus TSA). Western blot analysis showed that TSA increased the levels of GR proteins in the hippocampi of MS rats (Fig. 6c; *t* = 3.592, *P* = 0.008 DMSO versus TSA).

### TSA reversed the effect of MS on the activation of NF-κB signaling and neuroinflammatory responses to sevoflurane

**Fig. 7:**
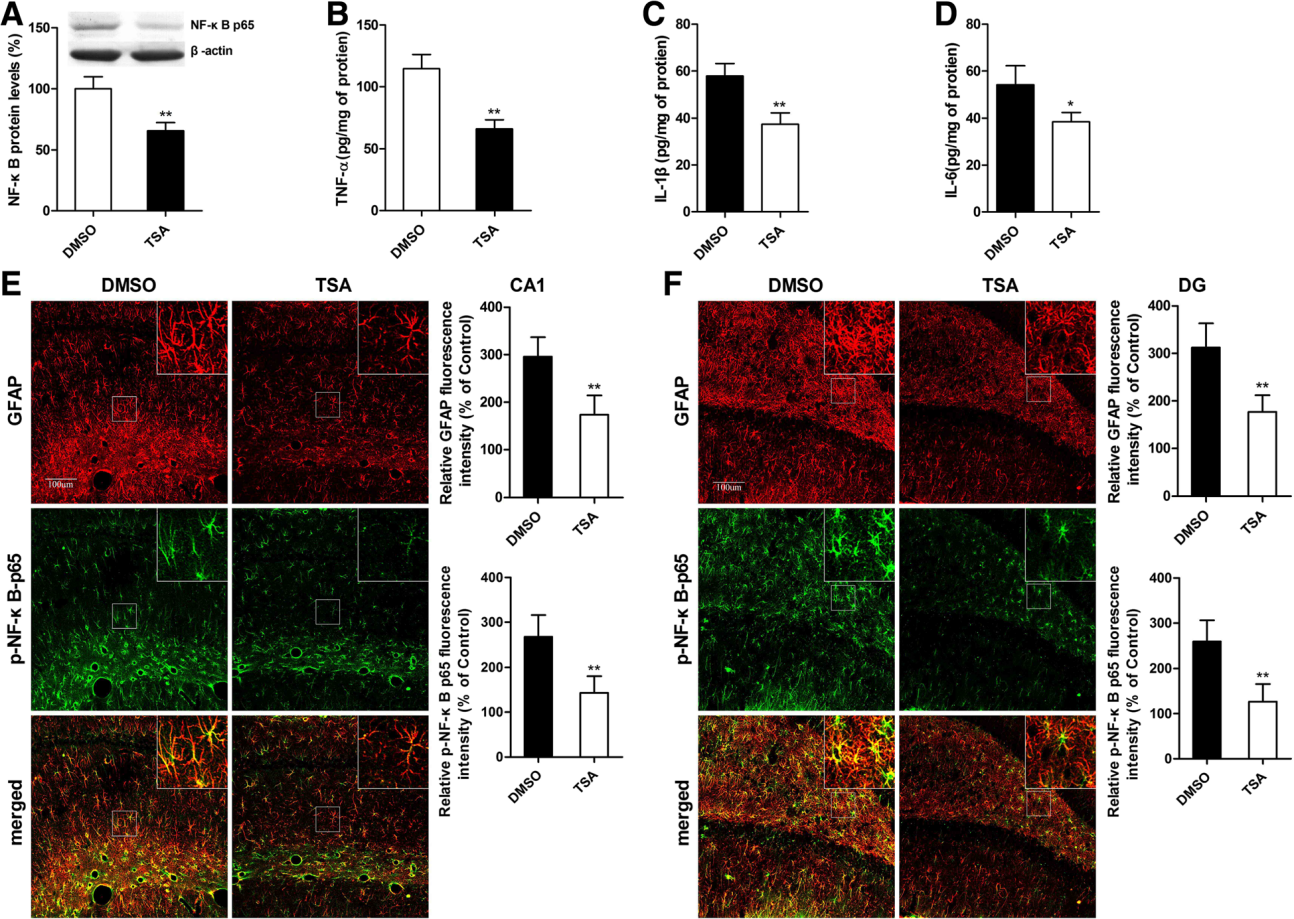
TSA reversed the effects of MS on the activation of NF-κB signaling and neuroinflammatory responses to sevoflurane in the hippocampal tissues in adult MS rats. **a** TSA pretreatment prevented the enhancement effect of MS on the levels of nuclear NF-κB p65 protein 12 h after anesthesia. **b–d** TSA pretreatment suppressed the increase in hippocampal TNF-α, IL-1β, and IL-6 levels 12 h after anesthesia in adult MS rats. **e** The fluorescence intensities of GFAP and p-NF-κB p65 in the TSA group rats were obviously lower than those in the DMSO group rats. Double immunofluorescence staining showed that p-NF-κB p65 (*green*) mainly co-localized (*merged*) with GFAP-positive reactive astrocytes (*red*) in the CA1 area of the hippocampus. **f** The changes in the fluorescence intensities of GFAP and p-NF-κB p65 in the DG area of the hippocampus were similar to those in the CA1 area of the hippocampus. ^*^ *P* < 0.05, ^**^ *P* < 0.01 versus DMSO. *Error bars* represent the means ± SD (*n* = 6). Statistical analyses were performed with Student’s two-sample *t* test

The effects of MS on the activation of NF-κB signaling and neuroinflammatory responses to sevoflurane seem to be partly associated with changes in the hippocampal GR levels. Given that TSA treatment reversed the hippocampal expression of GR in adult MS rats, we tested the activation of NF-κB signaling and neuroinflammatory responses to sevoflurane in TSA—and vehicle-treated MS rats. A volume of 2 μl of TSA (100 ng/ml in DMSO) was infused into the lateral ventricle of MS rats for seven consecutive days before exposure to sevoflurane. Western blot analysis showed that TSA pretreatment significantly prevented the enhancement effect of MS on the levels of nuclear NF-κB p65 protein 12 h after anesthesia in MS rats (Fig. 7a; *t* = 7.060, *P* < 0.001 DMSO versus TSA). An ELISA showed that TSA pretreatment also markedly suppressed the increase in hippocampal TNF-α, IL-1β, and IL-6 levels 12 h after anesthesia in adult MS rats (Fig. 7b–d; TNF-α, *t* = 8.564, *P* < 0.001 DMSO versus TSA; IL-1β, *t* = 6.954, *P* < 0.001 DMSO versus TSA; IL-6, *t* = 3.169, *P* = 0.001 DMSO versus TSA). The fluorescence intensity of GFAP in the TSA group rats was markedly lower than that in the DMSO group rats at 12 h after anesthesia (Fig. 7e, f; CA1, *t* = 3.581, *P* = 0.005 DMSO versus TSA; DG, *t* = 4.188, *P* = 0.002 DMSO versus TSA). A similar change in pattern was observed for phosphorylated NF-κB (p-NF-κB p65) (Fig. 7e, f).

### TSA attenuated the sevoflurane-induced cognitive impairment in adult maternal separation rats

**Fig. 8:**
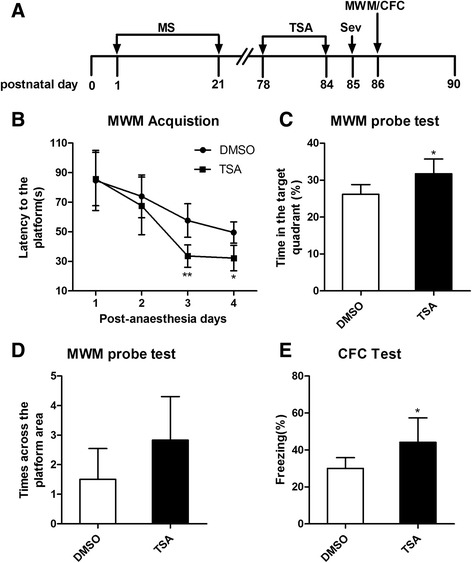
TSA attenuated sevoflurane-induced cognitive impairment in adult rats with neonatal maternal separation. **a** Schematic illustration of the experimental timeline. *MS* maternal separation, *Sev* anesthesia with 3% sevoflurane for 2 h, *TSA* trichostatin A, *MWM* Morris water maze, *CFC* context fear conditioning. **b** TSA group rats had a shorter escape latency than DMSO group rats on the third and fourth days after sevoflurane anesthesia. There was a statistically significant interaction of time and group between the different groups (^*^ *P* < 0.05). Analysis was performed by repeated-measures two-way ANOVA. **c** The time in the target quadrant in the TSA group was more than that in the DMSO group by the MWM probe test. **d** There was no significant difference in the number of crossings of the platform area between the two groups of rats. **e** TSA group rats exhibited increased freezing to context relative to DMSO group rats. ^*^ *P* < 0.05, ^**^ *P* < 0.01 versus DMSO. *Error bars* represent the means ± SD (*n* = 10). Statistical analyses were performed with Student’s two-sample *t* test

Finally, we evaluated whether the reversal of the maternal effect on GR expression and neuroinflammatory responses to sevoflurane could attenuate sevoflurane-induced cognitive impairment. TSA was infused into the lateral ventricle of MS rats for seven consecutive days (P78–P84) before exposure to sevoflurane. We conducted the MWM (P86–P90) and CFC tests (P86–P88) 24 h after sevoflurane anesthesia. A schematic illustration of the experimental timeline is shown in Fig. 8a. The comparison of the escape latency showed that there was a statistically significant interaction between time and group between the different groups in the acquisition phase of the MWM test according to repeated-measures two-way ANOVA (*F* = 5.926, *P* = 0.035). The factors of days (*F* = 27.439, *P* < 0.001) and groups (*F* = 6.441, *P* = 0.029) were statistically significant. The post hoc Bonferroni test showed that the DMSO group rats had a longer escape latency than the TSA group rats on the third and fourth days after sevoflurane anesthesia (P88 and P89) (Fig. 8b; post-anesthesia day 3, *P* = 0.003 DMSO versus TSA; post-anesthesia day 4, *P* = 0.011 DMSO versus TSA). The time in the target quadrant in the DMSO group rats was less than that in the TSA group rats (Fig. 8c; *t* = 2.875, *P* = 0.021 DMSO versus TSA). There was no significant difference in the number of times across the platform area between the two groups (Fig. 8d). The TSA group rats showed increased immobility (freezing) at P88 (Fig. 8e; *t* = 2.672, *P* = 0.032 DMSO versus TSA). The results of the MWM and CFC tests revealed that TSA pretreatment attenuated hippocampal-dependent memory impairment induced by sevoflurane in MS rats.

## Discussion

Sevoflurane is one of the most commonly used inhalational anesthetics for general anesthesia. We first found that sevoflurane anesthesia in adult (P85) MS rats induced cognitive impairment. Moreover, we found that anesthesia with 3% sevoflurane for 2 h induced cognitive impairment only in MS rats and not in normal rats (Fig. 1), which suggests that sevoflurane anesthesia-induced cognitive impairment has different selectivity in different individuals. These findings provide a possible explanation for the clinical observation that the occurrence of POCD shows distinct individual differences and suggest that individuals who have experienced early life adversity may have a predisposition to cognitive impairment after anesthesia.

Accumulating evidence suggests a pivotal role for neuroinflammation in the POCD process. Pro-inflammatory cytokine release and astrocyte activation were associated with a decline in cognitive performance in humans and animals [6–8]. Pro-inflammatory cytokines, such as TNF-α, IL-1β, and IL-6, can be released by activated astrocytes, triggering neuroinflammation and leading to cognitive dysfunction [9]. Higher concentrations of pro-inflammatory cytokines inhibit long-term potentiation and impair memory [47]. We found that 3% sevoflurane anesthesia for 2 h increased the levels of TNF-α, IL-1β, and IL-6 in the hippocampus after anesthesia in both normal and MS rats, but the degree of pro-inflammatory cytokines increase in MS rats was markedly higher than that in normal rats after anesthesia (Fig. 2). These data suggest that sevoflurane anesthesia in adult MS rats may cause cognitive impairment by inducing excessive neuroinflammation. Sevoflurane anesthesia also increased GFAP (the marker of astrocyte activation) immunofluorescence in the hippocampus after anesthesia in both normal and MS rats. The degree of increase in GFAP immunofluorescence in MS rats was higher than that in normal rats, similar to the change in pro-inflammatory cytokine levels (Fig. 3). These results further suggest that rats with different early life experiences may have different neuroinflammatory reactions to sevoflurane anesthesia.

Inhalation anesthetics have been shown to activate NF-κB signaling [39, 44]. NF-κB signaling plays a pivotal role in immune and inflammatory responses. Inactive NF-κB, a dimer of p50 and p65, remains in the cytosol. In response to diverse internal and external inflammatory stimuli, NF-κB p65 is phosphorylated and rapidly translocates to the nucleus [48, 49]. In the current study, we found that sevoflurane anesthesia activated hippocampal NF-κB signaling both in MS rats and normal rats, but the levels of nuclear NF-κB p65 protein and the fluorescence intensity of p-NF-κB p65 in the hippocampus in MS rats were significantly higher than those in normal rats (Figs. 2 and 3). These results suggest that NF-κB signaling in MS rats was overactivated under sevoflurane anesthesia. Next, we found that the pre-inhibition of NF-κB signaling by PDTC significantly suppressed excessive pro-inflammatory cytokine release after sevoflurane anesthesia in MS rats (Fig. 4). These results further suggest that the excessive activation of NF-κB signaling was involved in the increase of neuroinflammation after sevoflurane anesthesia in MS rats.

Given that glucocorticoid receptors (GRs) exert essential immunoregulatory and anti-inflammatory actions, the interaction between GRs and NF-κB signaling is of particular importance. In principle, GR signaling and NF-κB signaling are mutually antagonistic. Dysregulated GR signaling may enable exaggerated NF-κB signaling, ultimately leading to greater inflammatory responses. In the current study, a decreased expression of GR in MS rats was observed, and MS rats had a greater percentage of DNA methylation in the exon 1_7_ GR promoter region (Fig. 5). These findings are in agreement with those of other studies that showed that early life adversity results in greater methylation of the GR gene promoter and reduced GR expression in rodents and humans, with consequent increases in pro-inflammatory gene expression [29–34]. Therefore, we speculate that the MS phenotype was associated with the downregulation of GR and the increased production of pro-inflammatory cytokines controlled by the pro-inflammatory transcription factor NF-κB, which may worsen sevoflurane anesthesia-induced neuroinflammation and ultimately lead to cognitive impairment. A key question is whether the impact of early experiences on epigenetic programming is reversible in adults. It has been reported that DNA methylation produced by insufficient maternal care is reversible in the hippocampus of adult offspring through the modulation of the chromatin structure using the histone deacetylase inhibitor TSA [50]. Histone deacetylase prevents histone acetylation and ensures that histones bind tightly to DNA. The activation of chromatin through histone deacetylase inhibition might trigger DNA demethylation by increasing the accessibility of methylated DNA to demethylase activity [51]. We found that TSA decreased the percentage of DNA methylation in the exon 1_7_ GR promoter region. Then, marked increases in the hippocampal GR expression were accompanied by the reversal of DNA methylation (Fig. 6).

Finally, we assessed the effects of the reversal of GR expression in adult MS rats on the activation of NF-κB signaling and neuroinflammatory responses to sevoflurane anesthesia. TSA pretreatment significantly suppressed overactivated NF-κB signaling and excessive neuroinflammation (Fig. 7). Moreover, TSA pretreatment ameliorated sevoflurane anesthesia-induced cognitive impairment in adult MS rats. These results suggested that enhanced GR expression may suppress the inflammatory response and prevent cognitive impairment induced by sevoflurane anesthesia. Taken together, our findings support the conclusion that early life adversity downregulates the expression of GR, enhances NF-κB signaling, and worsens sevoflurane anesthesia-induced neuroinflammation, ultimately leading to cognitive impairment.

There are several limitations and caveats that should be considered in this study. First, we did not systematically investigate the effects of MS on the function of the hypothalamic–pituitary–adrenal (HPA) axis and instead focused on the expression of GR in the hippocampus, which is closely linked to HPA axis function [52]. It is worth noting that higher cortisol levels and ineffective dexamethasone treatments have been reported in POCD patients [53, 54]. However, the current results suggested that the MS-induced downregulation of GR expression in the hippocampus may amplify neuroinflammation and cause cognitive impairment, which will allow us to further investigate the association between early life adversity, the HPA axis, and POCD. Second, MS can induce DNA hypermethylation in other genes (e.g., brain-derived neurotrophic factor) in addition to the GR gene, and the demethylation function of TSA is not specific to the GR gene [55, 56]. More research is needed to determine whether other mechanisms of early life adversity are involved in cognitive impairment after anesthesia. However, given the inflammatory mechanisms of POCD, the epigenetic regulation of GR is most likely the chief reason for MS-induced cognitive impairment after anesthesia. Finally, volatile anesthetics seem to have a confounding effect on the brain. Some beneficial effects have been reported for isoflurane on the outcome of cerebral ischemia-reperfusion injury, traumatic brain injury, and lipopolysaccharide-induced cerebral damage [57–59]. Perhaps volatile anesthetics have a neuroprotective effect on brain injury. However, volatile anesthetics themselves may have adverse effects on the brain. Recent studies show that volatile anesthetics can cause neuroinflammation and impairment in a normal physiological state [10, 11].

## Conclusions

We found that early life adversity may be associated with increased vulnerability to cognitive decline after anesthesia. These findings suggest that the downregulation of GR by MS enhanced NF-κB signaling and sevoflurane anesthesia-induced neuroinflammation, which plays an important role in cognitive impairment. In addition, the regulation of GR expression in the hippocampus may be one strategy to prevent anesthesia-induced cognitive impairment under certain conditions. This study provides insight into the mechanisms by which early life adversity produces a future risk of POCD. In future studies, it will be important to further determine whether early life adversity is an independent risk factor for POCD in clinical practice, ultimately leading to safer anesthesia and better postoperative prognoses for people who have experienced early life adversity.

## Abbreviations

CFC: Context fear conditioning
ELISA: Enzyme-linked immunosorbent assay
GFAP: Glial fibrillary acidic protein
GR: Glucocorticoid receptor
IL-1β: Interleukin1β
IL-6: Interleukin6
MS: Maternal separation
MWM: Morris water maze
NF-κB: Nuclear factor-kappa B
PDTC: Pyrrolidine dithiocarbamate
POCD: Postoperative cognitive dysfunction
TNF-α: Tumor necrosis factor-α
TSA: Trichostatin A
